# Is molecular allergology cost-effective and cost saving in children with suspected peanut allergy compared to double blind placebo controlled food challenge (DBPCFC), open oral food challenge and skin prick test in Sweden?

**DOI:** 10.1186/2045-7022-3-S3-P124

**Published:** 2013-07-25

**Authors:** S Glaumann, L-L Hermansson, B Mascialino, G Hubben, MP Borres, C Nilsson

**Affiliations:** 1 Department of Clinical Science and Education, Södersjukhuset, Karolinska Institutet, Stockholm, Sweden; 2 Thermo Fisher Scientific, Uppsala, Sweden; 3 BaseCase Software, Berlin, Germany; 4 Department of Paediatrics, Sahlgrenska Academy of Gothenburg University, Gothenburg, Sweden

## Background

Peanuts are one of the most common foods causing allergic reactions in children. IgE-ab sensitization to peanut has been reported in 7–11% of children in Western countries and the prevalence of peanut allergy (PA) in children varies between 0.75% and 3%. Given the PA impact on quality-of-life (QoL), accurate diagnosis is crucial because many sensitized individuals are actually tolerant to peanut. Peanut sensitization established by IgE antibodies (IgE-ab) in blood or skin prick test (SPT) often needs to be confirmed by the “gold standard” Double-blind placebo-controlled food challenge (DBPCFC), a risky and expensive procedure. In clinical practice an open oral food challenge (OC) is performed instead of a DBPCFC. PA can be effectively diagnosed using molecular allergology (MA), identifying subjects at risk for PA reactions (IgE-ab to Ara h 1-2-3). No cost-effectiveness (CE) analyses are available on MA for allergy.

## Methods

Three 5-year Markov models simulate the flow of 200 children PA suspected presenting to the general practitioner. The models compare different diagnostic approaches (DBPCFC, OC, SPT and MA), computing the cost-per-QALY (Quality Adjusted Life Year) gained based on data from the literature. Calculations were performed for Sweden and BaseCase® was used to present results. Care giver indirect costs are included in a sensitivity analysis.

## Results

In Sweden, cost-per-QALY is 3.66 for SPT, 3.22 for DBPCFC, 2.23 for OC and 4.34 for MA. The cost for different diagnostic approaches is:

- SPT: 44851 SEK

- DBPCFC: 24278 SEK

- OC: 33031SEK

- MA: 11267 SEK

## Conclusion

In Sweden, MA increases QoL, and it is associated with reduced costs per patient with respect to the other strategies. The hypothesized usage of MA could be a valid alternative and a useful diagnostic tool replacing the “gold standard” DBPCFC in selected cases, DBPCFC still being useful in subjects with conflicting immunological/clinical results.

## Disclosure of interest

S Glaumann: None declared, L-L Hermansson: Employee of Thermo Fisher Scientific, B Mascialino: Employee of Thermo Fisher Scientific, G Hubben: Employee of BaseCase Software, M Borres: Employee of Thermo Fisher Scientific, C Nilsson: None declared.

